# Comparison of physiological and behavioral nutrition-related factors in people with and without adolescent idiopathic scoliosis, from cohort data at 8 to 20 years

**DOI:** 10.1093/jbmrpl/ziad013

**Published:** 2024-01-04

**Authors:** Phoebe T T Ng, Kylie Tucker, Syeda Farah Zahir, Maree T Izatt, Leon Straker, Andrew Claus

**Affiliations:** The University of Queensland, Laboratory for Motor Control and Pain Research, School of Biomedical Sciences, St. Lucia, 4072, QLD, Australia; KK Women’s and Children’s Hospital, Physiotherapy Department, 229899, Singapore; The University of Queensland, Laboratory for Motor Control and Pain Research, School of Biomedical Sciences, St. Lucia, 4072, QLD, Australia; The University of Queensland, Centre for Health Services Research, Faculty of Medicine, Woolloongabba, 4102, QLD, Australia; Queensland University of Technology at the Centre for Children’s Health Research, Biomechanics and Spine Research Group, South Brisbane, 4101, QLD, Australia; Curtin University, School of Allied Health, Perth, 6102, WA, Australia; The University of Queensland, School of Health and Rehabilitation Sciences, St. Lucia, 4072, QLD, Australia; Royal Brisbane and Women’s Hospital, Tess Cramond Pain and Research Centre, Herston, 4029, QLD, Australia

**Keywords:** adolescent idiopathic scoliosis, Raine study, nutrition, BMI, BMD

## Abstract

Nutrition-related variables including lower body mass index (BMI), lower bone mineral density (BMD), altered body composition and hormone levels have been reported in adolescent idiopathic scoliosis (AIS). The aims of this study were to determine if physiological and behavioral nutrition-related factors differ between people with and without AIS, and to quantify their relationship with AIS, in unbiased cohort sample. BMI, presence of an eating disorder, leptin, adiponectin, BMD, vitamin D, lean mass, and fat mass were compared between those with and without AIS at ages 8, 10, 14, 17, and 20 years, and multiple logistic regression was performed between these variables and AIS. Lower total body BMD (median, 1.0 g/cm^2^ vs 1.1 g/cm^2^; p = .03) and lean mass (median, 38.8 kg vs 46.0 kg; p = .04) at age 20 years were observed in those with AIS compared to those without scoliosis. At age 20, the odds of AIS were 3.23 times higher for adolescents with an eating disorder compared to those with no eating disorder (95% CI, 1.02–8.63) when adjusted for BMI. Every 1 kg/m^2^ increase in BMI decreased the odds of AIS by 0.88 times (95% CI, 0.76–0.98), after adjusting for eating disorder diagnosis. In conclusion, lower BMI in mid-adolescence and presence of eating disorder outcomes, lower BMD, and lower lean mass in late adolescence were associated with the presence of AIS. Current data do not explain the mechanisms for these associations but suggest that serum leptin, adiponectin, and vitamin D are unlikely to be contributing factors. Conclusive determination of the prevalence of eating disorders in AIS will require further studies with larger sample sizes.

## Graphical Abstract

**Graphical Abstract:**
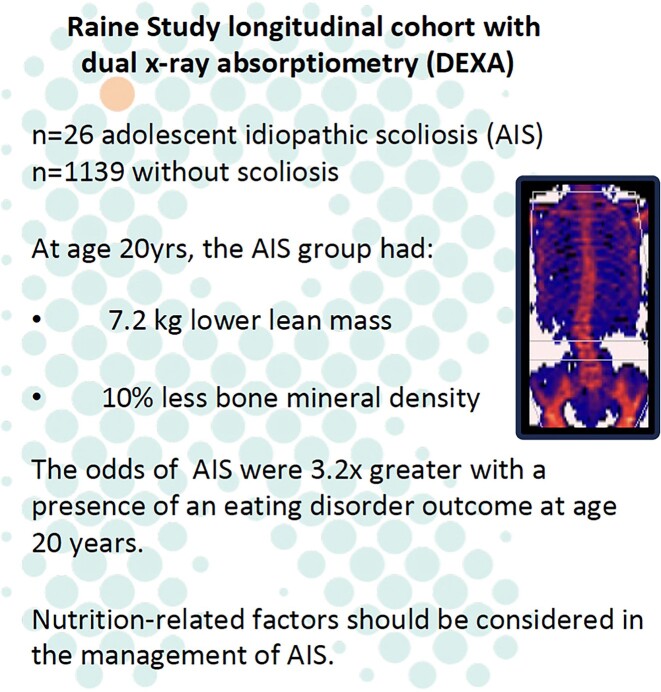

## Introduction

Nutrition-related factors may be important to understand with regard to both the etiology and clinical management of adolescent idiopathic scoliosis (AIS), which has been associated with lower body mass index (BMI).^1-3^ In a 2013 study by Ramirez et al,^2^ more than half (55.6%) of those with AIS were within the malnourished range (BMI < 18.5 kg/m^2^). This suggests the need to explore other nutrition-related factors that affect weight, such as eating disorders and appetite-regulating hormones.

Eating disorders are severe and persistence disturbances in eating behaviors, which are assessed by tools such as clinical interviews^4^ and questionnaires.^5^^,^^6^ There is mixed evidence regarding reports of a greater presence of eating disorders in those with AIS than in those without,^4^ with some evidence of no difference between the groups.^5^

Leptin and adiponectin are hormones that can influence weight by affecting appetite and glucose uptake.^7^ Greater adiponectin levels^1^ and lower leptin levels^8^ have been reported in participants with AIS compared with healthy controls. In a 2014 longitudinal study, a lower leptin level was associated with a greater risk of developing AIS.^1^

In the presence of eating disorders and altered appetite-regulating hormones, the body may receive less nutrition, which can affect bone deposition and body composition.^9-11^ Bone mineral density (BMD) is used as a measure of bone strength and an indicator for the risk of bone fractures.^12^ Lower BMD in those with AIS compared with those without has been reported.^13^^,^^14^ As lower levels of vitamin D are correlated with lower BMD, it may therefore be important to consider vitamin D levels in those with AIS.^15^ Indeed some evidence supports the presence of lower vitamin D levels in those with AIS compared with those without.^16^^,^^17^

There are mixed reports of a difference in body composition (distribution of fat, muscle, bone, and fluids) between those with and without AIS. Using a variety of tools such as bioelectrical impedance^2^^,^^18^ and dual energy X-ray absorptiometry (DXA),^1^ those with AIS have been reported to have lower body fat mass,^1^^,^^2^^,^^18^ skeletal muscle mass,^18^ and lean mass^1^^,^^2^ than those without AIS.

In summary, a pattern of poorer nutrition-related factors has been identified in those with AIS compared with healthy adolescents without scoliosis. Other than a UK cohort study,^1^ in the studies referenced above participants with AIS were patients recruited from a clinic and may have unknown confounding factors due to selection bias.^19^ For example, it is possible that adolescents who have a lower BMI have more obvious postural irregularities and therefore are more likely to present to the clinic. The potential for selection bias can be minimized by using data obtained from longitudinal cohort studies.^20^^,^^21^ In such studies, scoliosis can be identified from a large cohort using DXA scans rather than presentation at a clinic.

In a longitudinal cohort study (the Raine study), DXA scans at age 20 were used to identify adolescents with AIS (*n* = 26) and without scoliosis (*n* = 1139).^21^ The current study uses the same dataset and aimed to investigate the relationship between nutrition-related physiological (BMI, leptin, adiponectin, DXA-measured body composition, and vitamin D) and behavioral (presence of eating disorders) factors with AIS, cross-sectionally in an Australian cohort at ages 8, 10, 14, 17, and 20 years. We acknowledge that nutrition is profoundly influenced by a wide range of factors, such genetics, diet, gastrointestinal function, gut microbiota, and behaviors such as physical activity and sleep, but the scope of our study was to explore the particular behavioral drivers and the physiological consequences of nutrition, with the data that were available within the larger Raine cohort study.

## Materials and methods

### Recruitment

**Figure 1:**
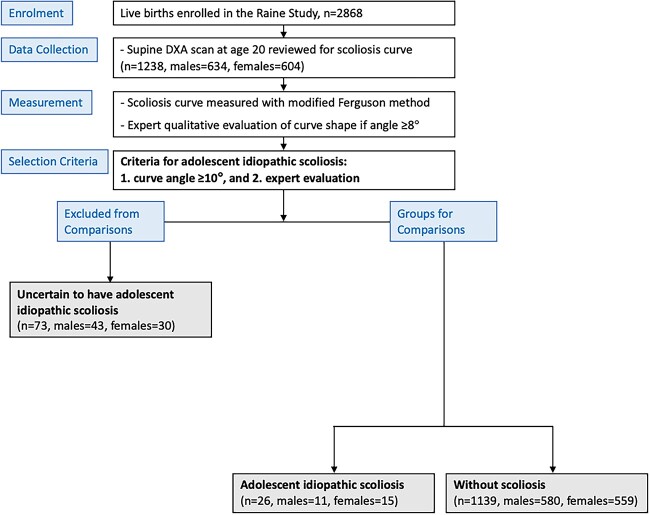
Consolidated Standards of Reporting Trials (CONSORT) diagram to detail the recruitment and allocation of participants for this study (adapted from Ng et al^21^). Participants were allocated into (1) adolescent idiopathic scoliosis and (2) without scoliosis and (3) uncertain to have adolescent idiopathic scoliosis based on their curve angle (measured modified Ferguson angle ≥10°) and expert evaluation.

This study used data from participants in the Raine study (www.rainestudy.org.au). This longitudinal cohort recruited 2868 mothers (Generation 1) and their children (Generation 2) born between 1989 and 1991, with subsequent collection of a wide array of biological, psychological, and social variables. These data have proven to be an unbiased sample, representing the general population in Western Australia.^22^ For the current study, 1238 participants from the same Raine study dataset underwent DXA scans, where we included adolescents with likely AIS (*n* = 26) and without scoliosis (*n* = 1139), and excluded those we were uncertain had AIS (*n* = 73) (Figure 1), that were identified in previous work.^21^ We compared 12 variables between participants with likely AIS and participants without scoliosis at ages 8, 10, 14, 17, and 20 years cross-sectionally. In addition, we explored the relationship between various nutrition-related physiological and behavioral factors with the presence of AIS. Consent for such use of these data was provided by the parents/guardians of the participants (ages 8–17 years) and by the participants themselves (age 20 years). This study was approved by the Raine Study Scientific Review Committee, and ethics approval was obtained from the Human Research Ethics Committee of The University of Western Australia.

### Groups

Generation 2 Raine study participants with AIS were determined using quantitative curve angle measurement of a scoliosis curve angle ≥10° and qualitative expert examination of curve shape from DXA scans at age 20 years without an official medical diagnosis, as previously reported.^21^ Those who reported a diagnosis of juvenile scoliosis at age 5 or 8 years (*n* = 2) were excluded prior to the analysis of curve angle and curve shape. From a total of 1238 participants, 26 (female = 15, male = 11) were determined to have AIS with a mean (SD) modified Ferguson angle (ie, scoliosis curve angle measured on DXA scans) of 14.0° (3.5°), which ranged from 10° to 24°. From the remaining participants, 1139 (females = 559, males = 580) were determined to be without scoliosis (Figure 1). To avoid including people with non-AIS causes of scoliosis, 73 were excluded from both groups because expert qualitative evaluation identified curves that were characteristic of measurement errors or other conditions despite having an angle ≥10° (qualitative criteria shown in Table 1).

**Table 1:** Qualitative criteria used by the expert reviewer for evaluation of spinal curves on DXA images, with the aim to identify likely adolescent idiopathic scoliosis.

**Description**	**Identifying features used**
Positional error	Obliquity of the pelvis that may produce an isolated lumbar curve
	Obliquity of the shoulders that may produce an isolated cervical or upper thoracic curve
	Participant generally not lying straight on scan, seen with lateral translation of the shoulders relative to the pelvis
Image shadow from internal organs	Unequal width of vertebrae in the midthoracic spine with the absence of any sort of compensatory curve above and/or below the pseudo-scoliosis, thought to be from an aortic arch and/or cardiac shadow
Other spinal pathology (non-idiopathic)	Acutely angulated spine over 2–4 spinal segments suggestive of scoliosis not of an idiopathic type or long C-shaped curve of spine suggestive of neuromuscular scoliosis
Consistent with idiopathic scoliosis	After exclusion of the above features, and participant has a lateral curve involving >4 spinal segments, in 1 of the common idiopathic scoliosis curve patterns (single thoracic/thoracolumbar/ lumbar or balanced thoracic and lumbar curves)

Reprinted with permission from Ng et al.^21^

### Body mass index

Body weight was measured to the nearest 0.1 kg with participants dressed in lightweight clothing, and height was measured with a hypsometer to the nearest 0.1 cm. Body mass index was calculated as weight divided by squared height (kg/m^2^) at 8, 10, 14, 17, and 20 years of age.

### Eating disorder questionnaire completed by the child

The Raine study eating disorder assessment items and diagnostic algorithms for determining DSM-5 (*Diagnostic and Statistical Manual of Mental Disorders, Fifth Edition*) eating disorders are outlined in detail in previous work.^23^^,^^24^ Assessment of eating disorder symptoms was adapted from the Child Eating Disorder Examination^25^ and also from the Eating Disorder Examination—Questionnaire.^26^ The adapted questionnaire was completed at ages 14, 17, and 20 years old. Diagnoses were based on responses to these items, combined with measured height and weight. This study investigated the eating disorder category and global score determined from this questionnaire. For the eating disorder category, results were dichotomized by grouping the full and partial eating disorder as “yes” (an eating disorder) and subthreshold and no disorder as “no” (no eating disorder). The eating disorder global score is a continuous, global index of eating disorder symptoms calculated by taking the mean of the items in the questionnaire.

### Clinician diagnosis of eating disorder reported by parent/child

The participants (ages 17 and 20) and their parents/guardians (participant’s age 17) answered “Do you have now or in the past, any of the following health professional diagnosed medical conditions or health problems?” For this analysis, the response “no” was categorized as not diagnosed and the responses “yes in the past,” “yes in the past and now,” or “yes, now” were categorized as diagnosed with an eating disorder.

### Serum leptin and adiponectin

Venous blood samples for serum leptin and adiponectin were taken from an antecubital vein after an overnight fast at 17 and 20 years. Laboratory assays were performed at an accredited central laboratory (Pathwest Laboratories, Perth, Australia). Leptin was measured by the ACTIVE Human Leptin ELISA kit (DSL-10-23 100; Diagnostic Systems Laboratories). Adiponectin was measured by the Quantikine Human Total Adiponectin/Acrp30 Immunoassay (R&D Systems).

### DXA absorptiometry measures

Whole-body scanning was performed at age 20, using DXA on a Norland XR-36 densitometer (Norland Medical Systems, Inc.), according to manufacturer-recommended procedures. The built-in machine software (version 4.3.0) provided estimates of whole-body BMD (g/cm^2^), fat mass (g), and lean mass (g). These scans were also used to identify participants with likely AIS, using quantitative and qualitative evaluation, as previously reported.^21^

### Vitamin D

To identify people at risk of vitamin D deficiency, serum 25-hydroxyvitamin D was measured. Fasting venous blood was collected at ages 14, 17, and 20 years. Serum was stored securely at −80°C. At 14 years, serum was measured using an enzyme immunoassay (Immunodiagnostic Systems Ltd.), and at ages 17 and 20, isotope-dilution liquid chromatography–tandem mass spectrometry was performed by RMIT Drug Discovery Technologies.^27^

### Statistical analysis

Descriptive statistics were used to summarize the baseline characteristics of the study population. Normality of distributions was assessed using Shapiro–Wilk’s test. Median and interquartile ranges are presented as data on most of the variables were not normally distributed. Categorical variables are presented using frequencies and percentages.

For between-group comparisons of continuous variables at each time point, normally distributed variables were assessed with *t* tests, and for nonnormal distributions, Mann-Whitney *U* tests were performed. Statistical differences between groups for categorical data at each time point were examined by Fisher’s exact test.

Logistic regression was conducted to explore the association of different predictors with the presence of AIS at each time point. Univariate logistic regression was first performed to examine the relationship between AIS and each explanatory variable. All variables with a p value < .2 in the univariate model were then considered in a multiple logistic regression model with stepwise selection (backwards elimination process). The combination of variables selected after the stepwise elimination process was included in the final multiple logistic regression model to assess their association when considered in combination. Statistical analyses were performed using Rstudio (version 2022.02.3; RStudio PBC ).

## Results

### Nutrition-related physiological measures

**Figure 2:**
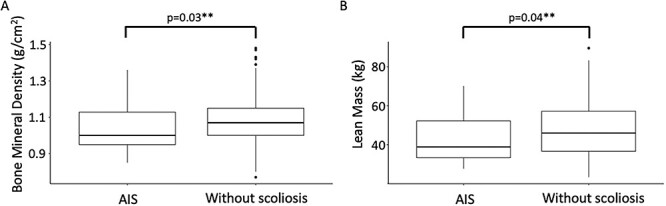
Boxplot distribution of (A) bone mineral density and (B) lean mass, at age 20 years, in those with adolescent idiopathic scoliosis (AIS) and without scoliosis. ^*^^*^p < .05.

**Figure 3:**
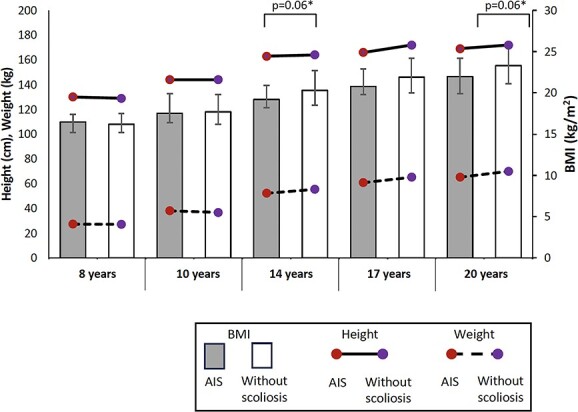
Distribution of medians of height, body weight, and body mass index (BMI) between 8 and 20 years old in those with adolescent idiopathic scoliosis (AIS) and without scoliosis. Error bars represent the interquartile range of BMI. ^*^p < .1 when comparing BMI between groups.

Descriptive (median and quartiles) and summary statistics by groups are presented in Tables 2 and 3. Those with AIS had a significantly lower total body BMD (median [IQR], 1.0 [0.2] vs 1.1 [0.5] g/cm^2^; p = .03) and lean mass (38.8 [18.8] vs 46.0 [20.7] kg; p = .04) than those without scoliosis at age 20 (Figure 2). For the other nutrition-related physiological variables—BMI (Figure 3), serum leptin, serum adiponectin, fat mass, and vitamin D levels—there were no statistically significant differences between groups (Table 2).

**Table 2:** Summary statistics of continuous outcomes by group at each time point.

	**AIS (*n* = 26)**					**Without scoliosis (*n* = 1139)**				
	**8 y**	**10 y**	**14 y**	**17 y**	**20 y**	**8 y**	**10 y**	**14 y**	**17 y**	**20 y**
Height, m	1.30 (1.26, 1.34) N = 21; p = .67	1.44 (1.39, 1.47) N = 20; p = .71	1.63 (1.59, 1.67) N = 21; p = .55	1.66 (1.63, 1.73) N = 20; p = .24	1.69 (1.64, 1.77) N = 26; p = .60	1.29 (1.25, 1.33) N = 1004	1.44 (1.25, 1.48) N = 981	1.64 (1.59, 1.70) N = 980	1.72 (1.65, 1.78) N = 874	1.72 (1.65, 1.79) N = 1137
Weight, kg	27.3 (25.1, 30.8) N = 21; p = .96	38.1 (33.3, 40.4) N = 20; p = .99	52.3 (49.2, 58.1) N = 21; p = .06^*^	60.9 (52.5, 67.4) N = 20; p = .05^*^^*^	65.3 (56.7, 72.9) N = 26; p = .04^*^^*^	27.2 (24.4, 30.6) N = 1003	36.7 (32.6, 42.6) N = 980	55.5 (49.6, 63.4) N = 981	65.2 (58.1, 74.0) N = 873	70.0 (61.2, 81.1) N = 1137
BMI, kg/m^2^	16.5 (15.2, 17.4) N = 21; p = .97	17.5 (16.4, 19.9) N = 20; p = .90	19.2 (18.2, 20.9) N = 21; p = .06^*^	20.8 (19.8, 22.9) N = 20; p = .11	22.0 (19.9, 24.2) N = 26; p = .06^*^	16.2 (15.2, 17.5) N = 885	17.7 (16.2, 19.8) N = 852	20.3 (18.5, 22.7) N = 980	21.9 (20.0, 24.2) N = 863	23.3 (21.1, 26.1) N = 1137
Eating disorder global score			0.34 (0.16, 0.70) N = 21; p = .81	0.44 (0.10, 1.15) N = 20; p = .58	0.45 (0.05, 0.91) N = 15; p = .84			0.34 (0.11, 0.68) N = 965	0.38 (0.06, 0.88) N = 842	0.36 (0.09, 0.73) N = 787
Serum leptin, μg/L				9.7 (1.9, 25.5) N = 18; p = .77	13.5 (3.1, 21.7) N = 22; p = .85				10.3 (2.7, 27.4) N = 845	9.0 (2.9, 22.1) N = 979
Serum adiponectin, mg/L				8.9 (7.2, 11.4) N = 18; p = .87	8.2 (6.2, 13.3) N = 22; p = .76				8.7 (6.0, 11.9) N = 845	9.1 (6.2, 13.0) N = 979
Vitamin D, nmol/L			74.7 (65.0, 84.7) N = 18; p = .50	67.3 (56.4, 92.5) N = 18; p = .83	77.1 (58.8, 85.8) N = 22; p = .50			77.6 (65.0, 91.3) N = 850	72.7 (57.9, 88.1) N = 845	71.5 (57.4, 85.8) N = 978
BMD, g/cm^2^					1.0 (0.95, 1.1) N = 26; p = .03^*^^*^					1.1 (1.0, 1.5) N = 1112
Fat mass, kg					20.1 (16.4, 28.7) N = 26; p = .96					20.0 (13.7, 27.9) N = 1112
Lean mass, kg					38.8 (33.3, 52.2) N = 26; p = .04^*^^*^					46.0 (36.6, 57.2) N = 1112

Abbreviations: AIS, adolescent idiopathic scoliosis; BMD, bone mineral density; BMI, body mass index; N, number of outcomes available.

Median values (first and third quartiles) and accompanying p values for each variable for those with AIS and without scoliosis at ages 8, 10, 14, 17, and 20 y are shown. Medians are reported as data were not normally distributed between groups; p values reported are between-group comparisons at each time point. ^*^p < .1; ^*^^*^p < .05.

**Table 3:** Summary statistics of categorical outcomes by group at each time point.

		**AIS (n = 26)**						**Without scoliosis (n = 1139)**					
		**Age 14 y**		**Age 17 y**		**Age 20 y**		**Age 14 y**		**Age 17 y**		**Age 20 y**	
		**Parent**	**Child**	**Parent**	**Child**	**Parent**	**Child**	**Parent**	**Child**	**Parent**	**Child**	**Parent**	**Child**
Eating disorder diagnosis (reported by clinician) (%)	Yes			4.4 N = 1 p = 1.0	0 N = 0 p = 1.0		0 N = 0 p = .62			5.3 N = 45	3.4 N = 30		5.3 N = 50
	No			95.6 N = 22	100 N = 22		100 N = 19			94.7 N = 802	96.6 N = 861		94.7 N = 887
Eating disorder diagnosis (questionnaire) (%)	Yes		4.8 N = 1 p = 1.0		19.0 N = 4 p = .12		25.0 N = 5 p = .07^*^		4.9 N = 49		8.9 N = 77		11.1 N = 105
	No		95.2 N = 20		81.0 N = 17		75.0 N = 15		95.1 N = 928		91.1 N = 793		88.9 N = 844

Abbreviations: AIS, adolescent idiopathic scoliosis; N, number of outcomes available.

The percentage of participants with an eating disorder diagnosis via report and questionnaire in those AIS and without scoliosis are shown. Eating disorder diagnosis was not assessed before 14 years of age; p values reported are between-group comparisons at each time point. ^*^p < .1.

### Nutrition-related behavioral measures

An eating disorder diagnosis based on the eating disorder questionnaire at age 20 showed a trend for higher prevalence in those with AIS compared with those without (25.0% vs 11.1%; p = .07), but this trend was not seen at younger ages (Table 3). For the eating disorder global score (Table 2) and the reported eating disorder diagnosis (Table 3), there were no statistically significant differences between groups.

### Results of logistic regression: relationship of variables with AIS

At age 14, based on stepwise methods, only BMI was selected in the final model as a predictor of AIS. Results suggested that, for every 1-kg/m^2^ increase in BMI, the odds of AIS decreased by 0.86 times (odds ratio, 0.86; 95% CI, 0.72–0.99; p = .06) (Table 4).

**Table 4:** Univariate and multiple logistic regression modelling of variables associated with AIS at each time point with estimate of odds ratios, 95% CIs, and p values.

	**Odds of AIS**					
	**Univariate logistic regression**			**Multiple logistic regression**		
	**Odds ratio**	**95% CI**	**p**	**Odds ratio**	**95% CI**	**p**
*Age 8 y*						
Height (m)	1.02	0.94–1.06	0.67			
Weight (kg)	0.99	0.90–1.06	0.74			
BMI (kg/m^2^)	0.95	0.74–1.13	0.56			
*Age 10 y*						
Height (m)	0.99	0.92–1.06	0.71			
Weight (kg)	0.99	0.93–1.04	0.62			
BMI (kg/m^2^)	0.98	0.83–1.12	0.77			
*Age 14 y*						
Height (m)	0.98	0.93–1.04	0.55			
Weight (kg)	0.96	0.91–1.00	**0.05** ^*^^*^			
BMI (kg/m^2^)	0.86	0.72–0.99	0.06^*^	0.86	0.72–0.99	**0.06** ^*^
Vitamin D (nmol/L)	0.99	0.97–1.01	0.46			
Eating disorder global score	1.00	0.35–2.32	1.00			
Eating disorder diagnosis (questionnaire)	0.97	0.05–4.79	0.97			
*Age 17 y*						
Height (m)	0.97	0.92–1.02	0.24			
Weight (kg)	0.96	0.91–1.00	**0.04** ^*^^*^			
BMI (kg/m^2^)	0.88	0.75–1.01	**0.09** ^*^			
Vitamin D (nmol/L)	1.00	0.98–1.02	0.74			
Eating disorder global score	1.34	0.64–2.55	0.40	2.43	1.00–5.38	**0.04** ^*^^*^
Serum leptin (μg/L)	0.99	0.97–1.01	0.60	0.97	0.93–1.00	0.12
Serum adiponectin (mg/L)	1.00	0.90–1.06	0.95			
Eating disorder diagnosis (questionnaire)	2.42	0.68–6.74	0.12			
Eating disorder diagnosis (clinician–parent)	0.81	0.04–4.00	0.84			
Eating disorder diagnosis (clinician–child)	0	NS	0.99			
*Age 20 y*						
Height (m)	0.99	0.95–1.04	0.76			
Weight (kg)	0.97	0.94–1.0	**0.05** ^*^^*^			
BMI (kg/m^2^)	0.88	0.78–0.98	**0.03** ^*^^*^	0.88	0.76–0.98	**0.04** ^*^^*^
Vitamin D (nmol/L)	1.00	0.99–1.02	0.62			
Eating disorder global score	1.06	0.34–2.83	0.92			
Serum leptin (μg/L)	1.00	0.97–1.02	0.81			
Serum adiponectin (mg/L)	1.01	0.93–1.07	0.88			
BMD (g/cm^2^)	0.02	0–0.74	**0.04** ^*^^*^			
Fat mass (kg)	1.00	1.00–1.00	0.66			
Lean mass (kg)	1.00	1.00–1.00	**0.07** ^*^			
Eating disorder diagnosis (questionnaire)	2.68	0.86–7.07	**0.06** ^*^	3.23	1.02–8.63	**0.03** ^*^^*^
Eating disorder diagnosis (clinician–child)	0	NS	0.99			

Abbreviations: AIS, adolescent idiopathic scoliosis; BMD, bone mineral density; BMI, body mass index.

Height and weight were not included in the multiple logistic regression analysis as they are related to BMI. At age 8 and 10 y, only univariate regression could be undertaken with BMI. ^*^p < .1; ^*^^*^p < .05.

At age 17, the multiple logistic regression model identified that every unit increase in the eating disorder questionnaire score increased the odds of AIS by 2.43 times (odds ratio, 2.43; 95% CI, 1.00–5.38; p = .04) when adjusted for serum leptin. There was no effect of serum leptin on the presence of AIS (odds ratio, 0.97; 95% CI, 0.93–1.00; p = 0.12) (Table 4).

At age 20, the multiple logistic regression model suggested that the odds of AIS were 3.23 times higher in participants having an eating disorder diagnosis (questionnaire) than those without it (odds ratio, 3.23; 95% CI, 1.02–8.63; p = .03) when adjusted for BMI. Every 1-kg/m^2^ increase in BMI decreased the odds of AIS by 0.88 times (odds ratio, 0.88; 95% CI, 0.76–0.98; p = .04) when adjusted for eating disorder (eating disorder questionnaire) (Table 4).

## Discussion

This study provides new information to profile physiological and behavioral factors relating to nutrition in an unbiased sample of people from ages 8 to 20 years with and without AIS. At age 20, BMD and lean mass were lower in those with AIS. There were no significant differences between groups for BMI, reported clinician and questionnaire-based diagnosis of eating disorder, serum leptin, adiponectin, fat mass, or vitamin D. However, multiple logistic regression models demonstrated an association of lower BMI at age 14, greater eating disorder score at age 17, lower BMI at age 20, and the presence of the questionnaire-based eating disorder diagnosis at age 20 with an increase in odds of having AIS. These results provide valuable insight into potential nutrition-related differences for consideration with regard to the risk of developing AIS and clinical management of those with AIS.

### Trend of lower BMI in those with AIS

With regard to BMI, multiple logistic regression models demonstrated that every 1-kg/m^2^ increase in BMI at ages 14 and 20 (odds ratio, 0.86–0.88) decreased the odds of having AIS, which is similar to previous studies (odds ratio between 0.80 and 0.89) with a narrow 95% CI of less than 1.^1^^,^^3^ While the standing heights in both groups were similar between 8 to 20 years, from the age of 14 years body weight was clinically significantly lower (~4%–7%)^28^ and, hence, BMI was also trending lower in those with AIS, with p values of .06. The weight and BMI in those with AIS were consistently lower as the participants increased in age. This study is the first to report comparisons of BMI cross-sectionally at different time points. The trend of lower BMI in those with AIS compared with their peers from mid-adolescence may indicate that this may be a co-existence or a consequence of AIS, rather than a cause of the condition. However, we were not able to determine the cause of the lower BMI.

### Presence of eating disorder outcomes in those with AIS

The statistical test for a difference between questionnaire-based eating disorder diagnosis at age 20 in those with AIS compared with those without scoliosis had a p value of .07, suggesting a higher prevalence in those with AIS. Previous studies that also used an eating disorder questionnaire have either not identified a difference between groups^5^ or provided evidence of a smaller proportion of eating disorders in those with AIS compared with those without scoliosis.^6^ In this study, while the questionnaire-based eating disorder at age 20 suggested a higher proportion of eating disorder diagnosis in those with AIS compared with those without scoliosis, the reported clinician eating disorder diagnosis was not different between groups. This may be because, while the questionnaire can be used to identify an eating disorder in an individual, the reported clinician diagnosis could only capture those who sought care and had been officially diagnosed by a healthcare practitioner. Multiple logistic regression models demonstrated that a greater eating disorder score (at age 17) and presence of a questionnaire-based eating disorder diagnosis (at age 20) were associated with increased odds of the presence of AIS. To our knowledge, no previous studies have explored the association of eating disorder outcomes with the presence of AIS. As eating disorders are a psychiatric condition associated with poor quality of life and high mortality,^29^ it may be important for healthcare professionals to check for signs of eating disorders through the course of clinical follow-up for scoliosis surveillance and management in the adolescent years.

### No differences in leptin, adiponectin, vitamin D, and fat mass between those with and without AIS

Leptin, adiponectin, vitamin D, and fat mass levels have been previously found to differ between cohorts with AIS versus those without, but this was not supported by the current data. For example, previous studies have identified that those with AIS have lower levels of leptin^1^^,^^8^ and vitamin D.^16^^,^^17^ Our data may indeed reflect a true lack of difference as our sample was an unbiased sample that was not sourced from those who presented to a scoliosis clinic. As AIS is most commonly first detected through postural irregularities in the absence of school screening,^30^ and postural irregularities are more obvious when the child is lean and has a lower body weight, clinic-based samples may have a bias towards lean children. While our population sample of AIS avoided this selection bias, it is important to highlight that those with AIS in our sample also had curve angles of milder severity (mean modified Ferguson angle of 14°, ranging from 10° to 24°). In another similar longitudinal study in the United Kingdom that identified those with AIS from approximately 5000 DXA scans of 15-year-old adolescents, the sample also had a small mean curve angle (modified Ferguson angle of 15.0°) but reported a wider range of curve severity, with 4 out of the 177 participants identified to have AIS exhibiting curve angles ≥40°.^20^ In a follow-up study of the same UK population sample, lower leptin levels were associated with increased odds of having AIS.^1^ Another possible reason for the UK study being able to demonstrate a difference in appetite-regulating hormones is that it had a larger AIS cohort (*n* = 177 vs our 26 participants). With our study demonstrating no differences in several nutrition-related factors that were commonly thought to be associated with AIS, this highlights the need to consider the effect of eliminating selection bias in a larger sample to examine the true relationship of factors that may influence the development of AIS.

### Lower BMD and lean mass in those with AIS

Bone mineral density and lean mass had prior evidence of differences in cohorts with AIS versus those without, and this was supported by our current data. Those with AIS were found to have a lower BMD^13^^,^^31^^,^^32^ and lean mass^1^^,^^2^ compared with those without. While our study only reports total BMD, a systematic review of 17 studies reported that those with AIS had a lower BMD at several sites (eg, femur, lumbar spine) when compared with non-AIS controls.^33^ Another study demonstrated lesser osteocyte (bone-forming cell) count in the trabecular bone of patients with AIS, which correlated well with lower BMD compared with the non-AIS controls.^32^ The approximately 9% lower BMD identified in those with AIS in our sample could indicate that those with AIS are at a greater risk of fractures, as an intervention study in healthy controls demonstrated that a 1% increase in BMD resulted in a 3% reduction in fracture risk.^34^ A lower whole-body BMD (not only in the spine) may suggest lower bone strength and weakened spinal architecture may contribute to the development of AIS. Our study demonstrated no differences in other nutrition-related factors, which raises the questions as to what other potential factors may be influencing BMD and lean mass and whether their reduced levels may be due to an isolated systemic response in those with AIS. Observations of BMD throughout childhood and adolescence, prior to the development of AIS, may contribute further insights to the pathogenesis of AIS and identify individuals at risk of developing AIS.

### Limitations

The modified Ferguson method for scoliosis curve measurement on DXA scans was first used by the Avon Longitudinal Study of Parents and Children in the United Kingdom, and it was identified by the authors that the modified Ferguson method on supine DXA scans to identify AIS results in a smaller curve angle compared with the Cobb method on standing radiographs.^20^ Subsequently, the UK cohort study chose to use a scoliosis curve angle of 6° as a cutoff to increase the sensitivity for identifying AIS.^20^ We followed with a publication that used the modified Ferguson method with a curve angle of 10° as a cutoff and qualitative examination by a scoliosis expert (author M.T.I., with >25 years of experience with identifying scoliosis curves on radiographs) to increase the specificity of identifying those with AIS.^21^ In that same study, we identified good to excellent interrater reliability between 2 raters for 41 scans (intraclass correlation coefficient, 0.82; 95% CI, 0.71–0.89; p < .001).^21^ Using DXA scans to identify AIS is a novel application for screening, but it is not a clinical diagnostic tool. It is possible that a small number of Raine participants with AIS may not have been detected. This approach prioritized specificity over sensitivity for identifying AIS with DXA scans. The prevalence of AIS (*n* = 26 vs *n* = 1139) in the current study is consistent with most studies showing from 2% to 3% AIS prevalence worldwide.^35^ Although applying a 10° cutoff for identifying AIS was associated with the resulting modest sample size and risks some reduction in statistical power, it also helped to reduce the risk of false-positive cases confounding the findings. Correction for multiple comparisons was not conducted as this analysis was exploratory in nature. While our analysis identified differences in BMI, BMD, lean mass, and the presence of eating disorders between those with and without AIS, it is important to note that more subtle, yet potentially real, differences may exist. However, these differences might not have been discernible due to our sample size. Future longitudinal studies with larger AIS sample sizes would be ideal to test the hypotheses advanced from the current study findings.

## Conclusion

Lower BMI in mid-adolescence and the presence of eating disorder outcomes, lower BMD, and lower lean mass in late adolescence were associated with the presence of AIS. Current data do not explain the mechanisms for these associations, but suggest that serum leptin, adiponectin, and vitamin D are unlikely to be contributing factors. Further research is needed to replicate the current findings. Conclusive determination of the prevalence of eating disorders in AIS will require further studies with larger sample sizes.

## Data Availability

The data used to generate the results in this publication are available upon request as ethical restrictions exist and data were obtained from a third party. Readers and interested researchers may contact the Raine study to request the data.

## References

[1] Clark EM , Taylor HJ, Harding I, et al. Association between components of body composition and scoliosis: a prospective cohort study reporting differences identifiable before the onset of scoliosis. J Bone Miner Res. 2014;29(8):1729–1736. 10.1002/jbmr.2207 24616164

[2] Ramirez M , Martinez-Llorens J, Sanchez JF, et al. Body composition in adolescent idiopathic scoliosis. Eur Spine J. 2013;22(2):324–329. 10.1007/s00586-012-2465-y 22886589 PMC3555626

[3] Hershkovich O , Friedlander A, Gordon B, et al. Association between body mass index, body height, and the prevalence of spinal deformities. Spine J. 2014;14(8):1581–1587. 10.1016/j.spinee.2013.09.034 24332597

[4] Alborghetti A , Scimeca G, Costanzo G, Boca S. The prevalence of eating disorders in adolescents with idiopathic scoliosis. Eat Disord. 2007;16(1):85–93. 10.1080/10640260701773660 18175235

[5] Smith FM , Latchford GJ, Hall RM, Dickson RA. Do chronic medical conditions increase the risk of eating disorder? A cross-sectional investigation of eating pathology in adolescent females with scoliosis and diabetes. J Adolesc Health. 2008;42(1):58–63. 10.1016/j.jadohealth.2007.08.008 18155031

[6] Zaina F , Donzelli S, Lusini M, et al. Adolescent idiopathic scoliosis and eating disorders: is there a relation? Results of a cross-sectional study. Res Dev Disabil. 2013;34(4):1119–1124. 10.1016/j.ridd.2013.01.001 23357674

[7] Yadav A , Kataria MA, Saini V, Yadav A. Role of leptin and adiponectin in insulin resistance. Clin Chim Acta. 2013;417:80–84. 10.1016/j.cca.2012.12.007 23266767

[8] Qiu Y , Sun X, Qiu X, et al. Decreased circulating leptin level and its association with body and bone mass in girls with adolescent idiopathic scoliosis. Spine (Phila Pa 1976). 2007;32(24):2703–2710. 10.1097/BRS.0b013e31815a59e5 18007248

[9] Misra M , Klibanski A. Anorexia nervosa and bone. J Endocrinol. 2014;221(3):R163–R176. 10.1530/JOE-14-0039 24898127 PMC4047520

[10] Considine RV , Sinha MK, Heiman ML, et al. Serum immunoreactive-leptin concentrations in normal-weight and obese humans. N Engl J Med. 1996;334(5):292–295. 10.1056/NEJM199602013340503 8532024

[11] Achari AE , Jain SK. Adiponectin, a therapeutic target for obesity, diabetes, and endothelial dysfunction. Int J Mol Sci. 2017;18(6):1321. 10.3390/ijms18061321 28635626 PMC5486142

[12] Marshall D , Johnell O, Wedel H. Meta-analysis of how well measures of bone mineral density predict occurrence of osteoporotic fractures. BMJ. 1996;312(7041):1254–1259. 10.1136/bmj.312.7041.1254 8634613 PMC2351094

[13] Hung VWY , Qin L, Cheung CSK, et al. Osteopenia: a new prognostic factor of curve progression in adolescent idiopathic scoliosis. J Bone Joint Surg Am. 2005;87(12):2709–2716. 10.2106/JBJS.D.02782 16322621

[14] Cheng JC , Qin L, Cheung CS, et al. Generalized low areal and volumetric bone mineral density in adolescent idiopathic scoliosis. J Bone Miner Res. 2000;15(8):1587–1595. 10.1359/jbmr.2000.15.8.1587 10934658

[15] Patton CM , Powell AP, Patel AA. Vitamin D in orthopaedics. J Am Acad Orthop Surg. 2012;20(3):123–129. 10.5435/JAAOS-20-03-123 22382284

[16] Balioglu MB , Aydin C, Kargin D, et al. Vitamin-D measurement in patients with adolescent idiopathic scoliosis. J Pediatr Orthop B. 2017;26(1):48–52. 10.1097/BPB.0000000000000320 27089048

[17] Gozdzialska A , Jaskiewicz J, Knapik-Czajka M, et al. Association of calcium and phosphate balance, vitamin D, PTH, and calcitonin in patients with adolescent idiopathic scoliosis. Spine (Phila Pa 1976). 2016;41(8):693–697. 10.1097/BRS.0000000000001286 27064335

[18] Tam EMS , Liu Z, Lam TP, et al. Lower muscle mass and body fat in adolescent idiopathic scoliosis are associated with abnormal leptin bioavailability. Spine (Phila Pa 1976). 2016;41(11):940–946. 10.1097/BRS.0000000000001376 26656046

[19] Berk R . An introduction to sample selection bias in sociological data. Am Sociol Rev. 1983;48(3):386–398. 10.2307/2095230

[20] Taylor HJ , Harding I, Hutchinson J, et al. Identifying scoliosis in population-based cohorts: development and validation of a novel method based on total-body dual-energy x-ray absorptiometric scans. Calcif Tissue Int. 2013;92(6):539–547. 10.1007/s00223-013-9713-y 23456028

[21] Ng PTT , Straker L, Tucker K, Izatt MT, Claus A. Advancing use of DEXA scans to quantitatively and qualitatively evaluate lateral spinal curves, for preliminary identification of adolescent idiopathic scoliosis. Calcif Tissue Int. 2023;112(6):656–665. 10.1007/s00223-023-01075-2 36907926 PMC10198858

[22] Straker L , Mountain J, Jacques A, et al. Cohort profile: the western Australian pregnancy cohort (Raine) study–generation 2. Int J Epidemiol. 2017;46(5):1384–1385j. 10.1093/ije/dyw308 28064197 PMC5837608

[23] Allen KL , Byrne SM, Oddy WH, Crosby RD. DSM-IV-TR and DSM-5 eating disorders in adolescents: prevalence, stability, and psychosocial correlates in a population-based sample of male and female adolescents. J Abnorm Psychol. 2013;122(3):720–732. 10.1037/a0034004 24016012

[24] Allen KL , Crosby RD, Oddy WH, Byrne SM. Eating disorder symptom trajectories in adolescence: effects of time, participant sex, and early adolescent depressive symptoms. J Eat Disord. 2013;1(1):32. 10.1186/2050-2974-1-32 24999411 PMC4081731

[25] Bryant-Waugh RJ , Cooper PJ, Taylor CL, Lask BD. The use of the eating disorder examination with children: a pilot study. Int J Eat Disord. 1996;19(4):391–397. 10.1002/(SICI)1098-108X(199605)19:4391::AID-EAT63.0.CO;2-G 8859397

[26] Fairburn CG , Beglin SJ. Assessment of eating disorders: interview or self-report questionnaire? Int J Eat Disord. 1994; 16(4):363–370. 10.1002/1098-108X(199412)16:4363::AID-EAT22601604053.0.CO;2-# 7866415

[27] Maunsell Z , Wright DJ, Rainbow SJ. Routine isotope-dilution liquid chromatography-tandem mass spectrometry assay for simultaneous measurement of the 25-hydroxy metabolites of vitamins D2 and D3. Clin Chem. 2005;51(9):1683–1690. 10.1373/clinchem.2005.052936 16020493

[28] Lau DC , Douketis JD, Morrison KM, et al. 2006 Canadian clinical practice guidelines on the management and prevention of obesity in adults and children [summary]. CMAJ. 2007;176(8):S1–S13. 10.1503/cmaj.061409 PMC1839777 17420481

[29] Linardon J , Shatte A, Messer M, Firth J, Fuller-Tyszkiewicz M. E-mental health interventions for the treatment and prevention of eating disorders: an updated systematic review and meta-analysis. J Consult Clin Psychol. 2020;88(11):994–1007. 10.1037/ccp0000575 32852971

[30] Altaf F , Gibson A, Dannawi Z, Noordeen H. Adolescent idiopathic scoliosis. BMJ. 2013;346(1):f2508. 10.1136/bmj.f2508 23633006

[31] Cheng JC , Guo X, Sher AH. Persistent osteopenia in adolescent idiopathic scoliosis. A longitudinal follow up study. Spine (Phila Pa 1976). 1999;24(12):1218–1222. 10.1097/00007632-199906150-00008 10382248

[32] Cheng JC , Tang SP, Guo X, Chan CW, Qin L. Osteopenia in adolescent idiopathic scoliosis: a histomorphometric study. Spine (Phila Pa 1976). 2001;26(3):E19–E23. 10.1097/00007632-200102010-00002 11224874

[33] Li XF , Li H, Liu ZD, Dai LY. Low bone mineral status in adolescent idiopathic scoliosis. Eur Spine J. 2008;17(11):1431–1440. 10.1007/s00586-008-0757-z 18751741 PMC2583185

[34] Cummings SR , Karpf DB, Harris F, et al. Improvement in spine bone density and reduction in risk of vertebral fractures during treatment with antiresorptive drugs. Am J Med. 2002;112(4):281–289. 10.1016/S0002-9343(01)01124-X 11893367

[35] Negrini S , Donzelli S, Aulisa AG, et al. 2016 SOSORT guidelines: orthopaedic and rehabilitation treatment of idiopathic scoliosis during growth. Scoliosis Spinal Disord. 2018;13(1):1–48. 10.1186/s13013-017-0145-8 29435499 PMC5795289

